# Verbatim Report of the Extraordinary General Meeting

**Published:** 1920-11-13

**Authors:** 


### THE COLLEGE OF NURSING (Limited by Guarantee).

### Verbatim Report of the Extraordinary General Meeting.

Held in Barnes Hall, Royal Society of Medicine, 1 Wimpole Street, London, W., on Thursday, November 4, 1920, the Hon. Sir Arthur Stanley (President) in the chair.

The Chairman: Ladies and Gentlemen,—The first thing I have to do is to propose to you that the minutes of the fifth annual general meeting be confirmed. These have all been printed and published, and I imagine you have all seen them. Is it your pleasure that they be taken as read and confirmed?—Agreed.

The Chairman: I now call upon the Secretary to read the notice convening the meeting.

### The Two Resolutions.

The Secretary (Miss M. S. Rundle) read the notice as follows: "Notice is hereby given that an extraordinary general meeting of the College of Nursing, Limited, will be held at the Royal Society of Medicine, 1 Wimpole Street, W. 1, on Thursday, the 4th day of November, 1920, at four o'clock in the afternoon, when the subjoined resolutions will be proposed:—

"1. That Article 6 of the Articles of Association be cancelled and the following Article substituted in its place:—

"6. A person appointed to be a member of the Council (including therein the President appointed by Clause 34 hereof) shall by virtue of such appointment become forthwith a member of the College. Except the subscribers hereto, no person shall be capable of being a member unless he be a member of the Council, or of a local Board, or be upon the Register. A person qualified under Section 3 (1) of the Memorandum of the Articles of Association may be placed upon the Register and become a member of the College by paying an entrance fee of one pound and one shilling, and by and on signing such form of application for registration and membership, and agreement to be bound by the Memorandum and Articles of Association and Regulations of the College, as the Council may from time to time require. Every member shall pay such annual subscription not being more than twenty shillings as the Council shall from time to time appoint. He may at any time pay to the Council such a sum as with the subscriptions already paid by him (not exceeding ten) equals twenty annual subscriptions at the date of such payment, and thereupon his annual subscriptions shall cease and he shall become a life member of the College. If his name be removed from the Register, he shall cease to be a member, but not otherwise.

"No member registered after November 20, 1920, shall enjoy any of the privileges of membership until he has paid his annual subscription and any arrears thereof.

"No member registered after November 20, 1920, shall vote at any annual meeting unless he has paid his annual subscription and any arrears thereof at least ten days before such annual meeting.

"The Council may order, if it shall think fit, the removal from the Register of the name of any member registered after November 20, 1920, who is in arrear with his subscriptions for five years or upwards.

"2. That the following words be added to Article 60, Clause 9:—and that at least twenty-eight days' previous notice of the meeting and its purpose shall be sent to the member with regard to whom an inquiry is to be held, and he shall have an opportunity of stating his case and defending himself before the Council.

"Should the above resolutions be passed by the requisite majority, they will be submitted for confirmation as special resolutions to a further extraordinary general meeting, which will be held on Saturday, the 20th day of November, 1920, at 3 p.m., at the Royal Society of Medicine, 1 Wimpole Street, W. 1.

"Dated the 6th day of October, 1920. By order of the Council."

The Chairman: Ladies and gentlemen,—Before calling upon Mrs. Jones to move the first resolution, I think perhaps it is just as well I should say two or three words about the reasons that have prompted us to this step. The real reason why this subscription is advocated lies in the success of the College itself. I think I described the College, at one of the annual meetings, as a very strong young infant. Well, that strong infant has not taken very long in outgrowing its clothes. We began, as you know, on a very modest scale. All of us who had anything to do with the foundation of the College were quite confident that the College was needed, and that it would be successful. We began it in the time of war, and it was difficult to foresee the future. I do not think any of us anticipated the success which the College has already achieved. Within the short space of about four years, to have got a membership of over 19,000—very nearly 20,000, I am told, the number we have always aimed at—to know that in those 19,000 we have got, as one of the Government officials described it to me the other day, "the very cream of the nursing profession," to have achieved that in four years I think is no small measure of success. But, of course, finance, in every walk of life, is, to a certain extent, a difficulty; and although I do not believe we ever thought we should be able to get £50,000 for the College, and not far short of £90,000 for the Tribute Fund which the College was instrumental in starting, to have got that in these difficult times is, I think, a very good augury of future success. But, of course, it is inevitable that our expenses should grow. During the first year they were comparatively small—so small that I remember when we wanted to equalise our budget I went to two or three private friends of my own and got them to make up the difference. But I am afraid that sort of thing is out of the question now, when our expenses amount to something not far short of £6,000 a year. That is one reason we are putting up the subscription.

### Reasons Given.

But another reason is that those first thousands, who, after all, were pioneers and were embarking on the unknown, they came forward and helped the movement at its outset; and it is not quite fair that the people who come in now and enjoy the fruits of the labours of others, of those pioneers, should come in on exactly the same terms. After all, when we began, they paid their guinea, and there was practically no return for it. Now, with all the activities of the College, you come into an establishment, into an established institution; and not only that, but new members will, we hope, soon come in to enjoy the benefits of a very beautiful building that has been given to us next door by the generosity of Lord and Lady Cowdray—(Applause)—so that all the educational work of the College, all the work that it is required to do, may be looked upon as that of the representative body of nurses. All that, of course, costs money. For that reason we are putting these resolutions before you to-day.

I would like to make one thing perfectly clear. It was never the intention of the Council of the College of Nursing, or of the members themselves, that this yearly subscription should be made obligatory on the members who joined the College before this date. Personally, I do not think it could have been done. The members who have joined up till now have done so on the definite understanding that one guinea was to qualify them as members of the College, and that is all we can ask of them. I think the resolution is perfectly clear, but a doubt has arisen in the minds of some nurses, and therefore, when Mrs. Jones moves it, she will do so in a way that makes that perfectly clear.

As to what the amount of the subscription shall be, that will be a question for you to deal with hereafter. I wish

**The College of Nursing—(*continued*).**

to repeat again—for it cannot be repeated too often—that anything given by any nurse who joined the College before this date will be, and must be, entirely voluntary: we have no intention of ever fixing an annual obligatory subscription upon them.

With regard to the other resolution, that is a question of what may be called that of the right of appeal. We took the right, of course, to strike a nurse's name off the Register; it is a right which is exercised by every voluntary body. But as it may possibly have frightened some nurses, so that they did not feel they wanted to join, we have modified it in this form, that she shall have an opportunity of stating her case and defending herself before the Council. And I think that meets practically the only criticism of our Articles of Association that I have yet seen. And I fervently hope that you will pass these resolutions, that all those members up to this date who can afford to give us an annual subscription will do so, and that the small annual subscription which we are going to put on is not likely to deter other nurses from joining. I now call upon Mrs. Jones to move the first resolution.

Mrs. Jones: Sir Arthur Stanley, ladies and gentlemen,—I have much pleasure in moving the resolution; but as the Secretary has received a notice of amendment to the resolution from a member, Miss Biggar, that after the words "Every member shall pay," etc., the words "Registered after November 20, 1920," shall be added, I have much pleasure in including these words in the resolution. It is my pleasure to propose the resolution in that way, as it makes it clear that the new regulation shall apply to those who join in the future. As Miss Biggar says, we cannot have the position stated too clearly. The resolution, therefore, reads: "That every member registered after November 20, 1920, shall pay such annual subscription, not being more than twenty shillings, as the Council shall from time to time appoint." (Applause.)

Miss Janet Macdonald: Mr. Chairman, ladies and gentlemen,—Speaking as one of the rank and file, and as one who, unfortunately, has not been able to take part in the life of the Centres, I have great pleasure in seconding this. And I would like to say that this great College is one which was much wanted, and in order to be a self-respecting profession we must be a self-supporting profession. I have much pleasure in seconding the resolution.

The Chairman: Does any lady or gentleman wish to make any remark before I put it? Then I will put it—that the resolution, as moved by Mrs. Jones and seconded by Miss Janet Macdonald, be adopted.—Carried unanimously, amid applause.

The Chairman: I now call on Miss Gibson to propose the second resolution.

Miss Gibson: I have great pleasure in proposing the second resolution, which, I think, must meet with the approbation of most of you. What I have to propose is, that added to Art. 60, Clause 9, there should be these words: "And that at least twenty-eight days' previous notice of the meeting and its purpose shall be sent to the member with regard to whom an inquiry is to be held, and he, or she, shall have the opportunity of stating his case and defending himself before the Council." It seems such an eminently just addition to the original resolution that I feel very fairly sure that it will meet with the approval of the whole meeting. It seems only fair. It appears to be the usual custom of English law to give an accused person the opportunity of defending himself, and it would be sad that anybody should be judged without being given that amount of justice which everybody has a right to expect.

A Member: Excuse me, but we could not hear the words of the resolution here.

The Secretary repeated the words.

Miss Slade: I have great pleasure in seconding this resolution.

The Chairman: Does anybody wish to ask any questions or make remarks on this? Then I put the resolution to the meeting.—Carried.

The Chairman: The question for discussion now is as to the voluntary annual subscription of foundation members. I say again—it cannot be repeated too often—that we cannot force any founder members to subscribe more; we want to know simply what they are prepared to give. (Laughter.)

Miss Goode (Matron of Otham Convalescent Home, Maidstone): May I be allowed to speak, as one of the older nurses and as one who joined the College of Nursing at first? I would like to ask that the subscription be limited to 5s. a year, and made quite definite. My reason for doing this is, perhaps, more on behalf of those of us who are getting on in years. Some of us found it a great effort to spare even the guinea to enrol ourselves as members. Since then our salaries have decreased largely in value, and so we are less able to afford things now than we were then. And, on the other hand, we do not like to accept benefits; we like to feel ourselves on a level with the coming generation, and that we are doing our part to help forward the College. I think we could afford 5s. a year, but anything further would cause distress amongst us, and therefore would be unacceptable. I would therefore like to propose that the subscription be limited to 5s. a year, that this be made quite definite, and that it be not altered without calling another meeting. I thank the Council for making even this subscription voluntary, and I think that appeals to all of us, and I would like to thank you very much for that consideration.

The Chairman: It might interest you if the Secretary were to read to you extracts from letters she has received on the point.

Opinions from all Quarters.

The Secretary (Miss Rundle):—

A agrees to a subscription, as she thinks it would be very much nicer for members to support their College themselves.

B agrees that all future members should pay an annual subscription, and most present members will want to do the same. Suggests a monthly *Bulletin*.

C agrees, and that the subscription should be on a sliding scale, according to the position of members.

D suggests that 10s. 6d. per annum would meet all pockets.

E personally glad to pay anything the meeting may decide upon. But for members who live afar off and cannot come to the Centres, could they be allowed to pay only 5s., which should not entitle them to the use of the Club Rooms, etc.? Members who use the Club Rooms should pay £1, which the writer considers a *very* small subscription compared with what most clubs require.

F quite willing to pay an annual subscription, whatever sum is named.

G in favour of an annual subscription; encloses 2s. 6d. until the amount is definitely fixed.

H has pleasure in sending her first annual subscription to the College, £1; does not know what the amount fixed may be, but feels that this year we ought to make a special effort as a thankoffering for what the College has done, and, if possible, to enter 20 Cavendish Square on a self-supporting basis.

I quite agrees that an annual subscription should be paid, and suggests 5s.

J strongly in favour of an annual subscription, whatever may be decided upon.

K would be glad to pay a small yearly subscription; the condition of the average nurse does not allow a large one, but if we ourselves are not reaping *immediate* benefit, it goes towards helping someone else, and no one wants to receive all and give nothing.

L in favour of an annual subscription. As members, we ought to be proud to maintain the College; it has done so much for us.

M pleased to give an annual subscription, whatever may be decided upon.

N was quite willing to afford £1 1s. fee to join the College when she was earning £35 per annum. Is now quite willing to pay whatever annual subscription should be considered

**The College of Nursing**—(*continued*).

necessary, even if it should be 20s. There are not many trained nurses who cannot afford to put by *less* than 6d. per week.

O would like to see graduated scale, for following reasons: (1) The different financial positions of members; (2) the opportunity of supporting the College as finances will allow.

P thinks it is our duty and just privilege to subscribe, if we mean to have an active College; hopes the fee will include *Bulletin*, which is such a great boon to country members.

Q suggests old members pay 10s. 6d., new members a guinea.

R suggests new members joining within six months of gaining certificate to pay 10s. 6d. per annum, and abolish extra fee for adding qualification to Register.

S suggests 5s. for new members; old members will give what they can. Sends 10s. Would like *Bulletin* monthly.

T quite agrees, but subscription must be kept as low as possible. Would like a monthly Bulletin.

U sends £1, and suggests it may do for two years.

V willing to pay a guinea, but suggests the principle of a sliding scale.

W thinks it behoves us to pay to keep up and to extend the work of the College.

X, a private nurse, would welcome the introduction of an annual subscription. The feeling of fellowship that the College gives is worth a great deal to a private nurse of some years' standing, who knows too well what it means to "stand alone."

Y sends a subscription, and promises early in the year to make up subscription for past years.

Z: To the College the nursing profession owes a very real debt of gratitude, and it is the duty of each member to endeavour to show appreciation by assisting financially.

In Opposition.

Then I have two letters from nurses who are not in favour of it.

A is against any other subscription than for Local Centre membership, as a great deal of money has been given to start and maintain the College, and few members can afford to pay; also a rumour has been started that untrained nurses are admitted. Is it true?

B does not think *nurses* can afford it; but as a Matron of a Co-operation the writer feels she *should* pay, but that the subscription should be collected from the Institution.

A Member: I agree with the last lady who spoke; I think 5s. definitely for the old members to pay a year would be on the right side, leaving it open to any of the older nurses who can afford to give something to give it to the Council, not as a subscription, leaving it open for them to do so. And as there are many private nurses and nurses in hospitals who have a dependence, some might afford £1, probably some more, according to our position in the nursing world. We have to think, not of ourselves, but of the majority of nurses who belong to the College. Some of them may be only getting £30 a year, while some may be receiving as much as £150 or £200 a year. I agree with the Matron who spoke that if you could agree to have 5s. a year from the older members it would be most acceptable to the majority of us. If we felt we could give more, so much the better for the Council.

Miss Goode: I made it clear, didn't I, that even the 5s. would be voluntary?

The Chairman: Yes. I have not been able to ascertain the views of the Council on this subject. I think it would be perfectly satisfactory if we could think that our 19,000 members, or even the greater part of them, would give 5s. each a year. That would give us not far short of £5,000 a year, and I think the Treasurer, Mr. Comyns Berkeley, would be satisfied if he got in £5,000 a year. Let us, then, make it clear. What will go out as a recommendation from this meeting is, that the existing members should be asked to pay a voluntary subscription of 5s. a year. (Applause.) And it should be made clear to them that, if they like to give more, they can do it as a donation, and we shall be very pleased to have it. Is that the decision of the meeting?—Carried unanimously.

The Secretary: There are one or two announcements to make—

Can Nurses Combine?

Lord Knutsford: I want to say one word about this College of Nursing. I want to say this because so many people have said to me, "What is the good of this College of Nursing? What is it doing? Why don't they do something for nurses?" Well, ladies, it is impossible for the College to do anything whilst it is quite so young. But I want all nurses in England to know that this is the very first time that any nurses have had the opportunity of voicing the feeling of nurses right throughout the country. Here is a College with a membership of 20,000 nurses, and we shall be every year a body to whom everybody who deals with nurses must listen, because if we can get the opinion of 20,000 nurses, or the majority of them, a Minister of Health, or anybody else, must listen to us. Therefore I do not want people to be impatient, and to say, "What has it done for nurses?" You may be quite certain that an organisation like this must act for the benefit of your profession. And then I have seen criticisms to this effect: "What business have you to have a man like that on? He is Chairman of the London Hospital; he is an employer of nurses, and therefore he does not represent nurses." That is not my feeling. My feeling is that I have been a slave of nurses for the last twenty-four years. (Laughter.) That I have been made to do what they want over and over again. I do not feel like an employer of nurses. I feel as a very humble servant of nurses. And if there is a feeling that because I happen to be the head of the London Hospital, or because my friend the Chairman is head of a hospital, we, as employers, should be kicked off the Council, it seems a pity. I think that, often, men who have been so closely associated with nurses know the wants of nurses almost as well as the nurses know them themselves, and perhaps they are in a position in the country to give voice to those nurses. Therefore I do not think we are altogether useless on the Council. I want you to go away from this meeting blessing the Council of the Nursing College, believing that at last we have somebody who can voice the wishes of nurses, and not to be too impatient if, in the first few years of our existence, we are not burning down London. (Laughter.) If we communicate with our members it costs £160 in postage. That is why you are asked to subscribe something.

Miss Cox Davies: We have heard a great deal about money this afternoon. I shall not say a word about money. I want, if I may, to propose, and ask you to carry, a vote of thanks to our Chairman for presiding over this meeting, and for all the splendid help that he gives, not only to the College, but, through the College, to the nursing profession. The College would never have come into existence if it had not been for the energy and determination of Sir Arthur Stanley. There is just one other name I would like to mention: we must not forget her, as she is not often with us now, and that is Miss Haughton, late Matron of Guy's, who helped us enormously in starting the College, and doing much earlier pioneer work in connection with it. I want you to carry a very hearty vote of thanks to our Chairman, who is an employer of nurses, to whom, perhaps, we look with reverence, and like just as much, if not more, just because he is an employer of nurses. (Applause.)

Miss Musson: I have much pleasure in seconding the resolution. I do not think I need add anything to what Miss Cox Davies has said; but I would like to make it clear that the Council of the College exists to serve the best interests of the nursing profession. (Applause.)—Carried with acclamation.

The Chairman: I must thank you very much for that vote of thanks. Miss Cox Davies said too much in assigning my share in founding the Nurses' College; the truth is, the College founded itself because it was badly wanted, and, like Topsy, it has grown. I think you will agree with me that Lord Knutsford looks remarkably well after twenty-four years of "slavery." He and I, as if being the slaves of

(*Continued on p.* 152.)

### The College of Nursing: Extraordinary General Meeting *(cont. from p. 157).*

our own matrons was not enough, have called into being a body of some twenty thousand nurses, which makes the matter much worse.

One thing which I want to say before you go is this. As you know, one-third of the Council retires every year. Now, it is important that nurses should take a share in the election of those who are going to represent the nursing profession. Frankly, the number of votes sent in last year and the year before was not very good, and I do hope that every nurse, every member of the College, will look upon it as part of her duty to try to get on the Council people who, they think, ought to represent the nursing profession. It is not fair to us if the voting papers are simply put into the waste-paper basket. We want to get at the head of the profession those who can voice the views of the profession and give effect to them.

Finally, I am authorised by Lord and Lady Cowdray to say that they propose to hand over this beautiful house in Cavendish Square, not only the house itself, but they propose to hand it over fully furnished and equipped. I believe it is hoped that it may be opened somewhere about next June as a complete going Club, and from what I have seen of some of the furniture, it will be one of the most beautifully furnished Clubs in the whole of London. I hope that you will all make a point of turning up on the opening day in order to show Lord and Lady Cowdray how very deeply and sincerely we appreciate their magnificent generosity. (Applause.)

Lord KNUTSFORD: It has always been a difficulty with me—how could the nurses know who to vote for? Of course there are men like Lord Knutsford who will always advertise themselves—(laughter)—and those are the sort of people you know. But how can 20,000 nurses scattered all over London know who to vote for? I think that is our difficulty in getting a vote. I do not wonder they light their pipes —no, cigarettes—with the papers. I want to know how nurses scattered all over England can possibly know; I do not wonder they do not vote, and I think that is the great difficulty in getting a ballot of nurses. The only people you can possibly know are those people who advertise themselves, as I do. Nurses do not vote because they do not know the people.

The CHAIRMAN: It is quite true we cannot all advertise ourselves as well as Lord Knutsford does—we know that; but I think what we have to try to do is to bring the College down to the members by instituting these centres. There is no earthly reason why a nurse should not take an active part in the centre near her own place, and through that centre she might know who to vote for, for at that centre they can perfectly well have meetings and discuss who they would like to have on the Council. If we did anything more, if we tried to say who they were to vote for, I think Lord Knutsford and others would be the very first to say we were interfering with the freedom of the subject, and I am perfectly certain he and I will be accused, as employers of labour, of manufacturing a Council to our own liking.

Lord KNUTSFORD: But how difficult it is, Sir Arthur, to get all the private nurses represented! At the present moment we have only the private nurses represented by one lady, who is head of the Nurses' Co-operation—a very large institution.

The CHAIRMAN: That is exactly what the private nurses themselves have got to combine to do.

Lord KNUTSFORD: They cannot combine; they are all over the world.

The CHAIRMAN: They have got to combine.

Lord KNUTSFORD: They cannot.

The CHAIRMAN: Well, Lord Knutsford says they cannot. It is up to the private nurses to show Lord Knutsford that they can. (Applause.)

Miss GOODE (Maidstone): I can only say, speaking as a country nurse stationed four miles from anywhere, that it is very difficult to combine with other people. You feel frightfully lonely, and you never hear anything about the College except what you get by post. We cannot often come up to London, even from Maidstone. This is the time of my life, because it is probably the only time I shall ever be heard here. (Laughter.)

The CHAIRMAN: If anybody can suggest anything that we can do, will you let us have the suggestion? We will try to carry it out.

Miss GERALDINE BREMNER: I should like to correct a statement Lord Knutsford made. I am not the head of the Nurses' Co-operation. I am merely one of the rank and file.

The CHAIRMAN: You cannot even be called an employer?

Miss BREMNER: No.

The CHAIRMAN: You are lucky. Next time we all meet I hope it may be in our new home. Thank you.

The proceedings then terminated.

